# Synergistic and targeted therapy with a procaspase-3 activator and temozolomide extends survival in glioma rodent models and is feasible for the treatment of canine malignant glioma patients

**DOI:** 10.18632/oncotarget.19085

**Published:** 2017-07-07

**Authors:** Avadhut D. Joshi, Rachel C. Botham, Lisa J. Schlein, Howard S. Roth, Antonella Mangraviti, Alexandra Borodovsky, Betty Tyler, Steve Joslyn, Jayme S. Looper, Michael Podell, Timothy M. Fan, Paul J. Hergenrother, Gregory J. Riggins

**Affiliations:** ^1^ Department of Neurosurgery, School of Medicine, Johns Hopkins University, Baltimore, MD, USA; ^2^ Department of Chemistry, University of Illinois Urbana-Champaign, Urbana, IL, USA; ^3^ Department of Pathobiology, University of Illinois Urbana-Champaign, Urbana, IL, USA; ^4^ VetCT Australia, Fremantle WA, Australia; ^5^ Department of Veterinary Clinical Sciences, Louisiana State University, Baton Rouge, LA, USA; ^6^ Department of Neurology, MedVet Chicago, Chicago, IL, USA; ^7^ Department of Veterinary Clinical Medicine, University of Illinois Urbana-Champaign, Urbana, IL, USA

**Keywords:** PAC-1, procaspase-3 activator, glioblastoma, small molecule therapy

## Abstract

**Purpose:**

Glioblastoma is a deadly brain cancer with a median survival time of ∼15 months. Ionizing radiation plus the DNA alkylator temozolomide (TMZ) is the current standard therapy. PAC-1, a procaspase-3 activating small molecule, is blood-brain barrier penetrant and has previously demonstrated ability to synergize with diverse pro-apoptotic chemotherapeutics. We studied if PAC-1 could enhance the activity of TMZ, and whether addition of PAC-1 to standard treatment would be feasible in spontaneous canine malignant gliomas.

**Experimental Design:**

Using cell lines and online gene expression data, we identified procaspase-3 as a potential molecular target for most glioblastomas. We investigated PAC-1 as a single agent and in combination with TMZ against glioma cells in culture and in orthotopic rodent models of glioma. Three dogs with spontaneous gliomas were treated with an analogous human glioblastoma treatment protocol, with concurrent PAC-1.

**Results:**

Procaspase-3 is expressed in gliomas, with higher gene expression correlating with increased tumor grade and decreased prognosis. PAC-1 is cytotoxic to glioma cells in culture and active in orthotopic rodent glioma models. PAC-1 added to TMZ treatments in cell culture increases apoptotic death, and the combination significantly increases survival in orthotopic glioma models. Addition of PAC-1 to TMZ and radiation was well-tolerated in 3 out of 3 pet dogs with spontaneous glioma, and partial to complete tumor reductions were observed.

**Conclusions:**

Procaspase-3 is a clinically relevant target for treatment of glioblastoma. Synergistic activity of PAC-1/TMZ in rodent models and the demonstration of feasibility of the combined regime in canine patients suggest potential for PAC-1 in the treatment of glioblastoma.

## INTRODUCTION

Gliomas are the most common type of malignant primary brain tumor, with ∼17,000 newly diagnosed cases each year in the United States [1]. Glioblastomas are now divided into two groups based on the presence or absence of mutations in the IDH1 or IDH2 genes [2]. Glioblastomas are the most aggressive and frequent of the malignant gliomas, accounting for ∼80% of all malignant gliomas [3]. Unfortunately, glioblastomas are uniformly rapid in their progression, and lethal; standard-of-care treatment with the DNA alkylator temozolomide (TMZ) and radiation yields a 2-year survival rate of 27% and a 5-year survival of <5% [4]. TMZ, in combination with surgical resection and focal radiation therapy, has been adopted as frontline therapy for glioblastoma, extending median patient survival from 12.1 months (radiation alone), to 14.6 months (radiation + TMZ) [4, 5].

As brain tumors, glioblastomas reside behind the defense of the blood-brain barrier, a protective physiological feature that precludes most chemotherapeutics from accessing the tumor, thereby severely limiting therapeutic options [6]. A further challenge in treating glioblastoma is its intrinsic resistance to apoptosis, through the overexpression of anti-apoptotic proteins such as Bcl-2 [7, 8], and other mechanisms. Analysis of primary tumor samples and cancer cell lines have shown procaspase-3 levels to be elevated in numerous cancer types [9]. Thus, although common hallmarks of cancers are malignant alterations that inhibit apoptotic signaling [10], these cells paradoxically express elevated levels of the executioner caspase responsible for carrying out the cellular proteolysis. While there appears to be little or no expression of procaspase-3 in most types of normal brain cells [11], limited analysis of glioblastoma cell lines and patient samples suggests robust expression of procaspase-3 in glioblastoma [7, 12–14]. In addition, because procaspase-3 is downstream in the apoptotic cascade from the typical anti-apoptotic alterations (such as Bcl-2 overexpression), this procaspase-3 elevation offers an opportunity for the selective induction of apoptotic cell death in glioblastoma.

PAC-1 is a small molecule that has been shown to facilitate the direct activation of procaspase-3 to caspase-3 [15–17], induce apoptotic death in many cancer types with elevated procaspase-3 levels [9, 15, 17], and PAC-1 and derivatives induce apoptotic death in a manner that is independent of the expression of Bcl-2 family proteins [18–20]. Further support for PAC-1's direct procaspase-3 activating mechanism of action comes from detailed studies of PAC-1 with cancer cells, evaluating the time dependence of procaspase-3 activation relative to the release of cytochrome c from the mitochondria or activation of upstream procaspases [15–17, 21]. PAC-1 facilitates the activation of procaspase-3 to caspase-3 through chelation of labile inhibitory zinc from procaspase-3 [22]. In addition to broad anticancer activity, PAC-1 penetrates the blood-brain barrier (BBB) [23]; thus the elevated procaspase-3 target combined with the unusual ability of PAC-1 to penetrate the central nervous system suggests the intriguing potential of PAC-1 for the treatment of glioblastoma. PAC-1 is currently being evaluated as a single-agent in a Phase I clinical trial in human cancer patients (NCT02355535).

Procaspase-3 activation for the treatment of gliomas is provocative as cellular concentrations of procaspase-3 in some glioblastoma patient samples have been quantified in the single digit micromolar range [12], elevated well above the ∼100 nM concentration believed to exist in normal cells [24]. Furthermore, a heightened latent level of caspase-3 activity has been observed in glioblastoma patient samples and has been linked to an increased malignant invasive phenotype [12, 14, 25]. This level of caspase-3 activity is insufficient for the execution of apoptosis, but suggests PAC-1 may be particularly effective in activating this partially primed pool of procaspase-3/caspase-3, thereby providing the greatest therapeutic benefit to the invasive glioblastoma cells remaining after surgery.

By relieving the physiologic labile zinc inhibition of procaspase-3, PAC-1 is able to enhance the ability of procaspase-3 to undergo auto-activation, sensitize the cellular procaspase-3 population to upstream apoptotic signaling events, and enhance the activity of caspase-3 generated through the induction of apoptosis [22, 26]. In recent reports PAC-1 has demonstrated dramatic synergy with a number of anticancer agents [27], most notably with doxorubicin for the treatment of metastatic osteosarcoma [26], and with vemurafenib for the treatment of mutant BRAF melanoma [28].

We now report the evaluation of PAC-1 for glioblastoma treatment in models of increasing complexity and clinical relevance. In particular, because TMZ exerts its anticancer activity through DNA damage and the subsequent induction of apoptosis [29], the combination of PAC-1 and TMZ was explored in detail. Here we show that PAC-1 and TMZ induce synergistic death in glioblastoma cells in culture and in rodent models, and combining PAC-1 with TMZ and ionizing radiation is well tolerated and associated with marked tumor regression in pet dogs with naturally-occurring glioma. These results support procaspase-3 activation as a mechanistically-based strategy to selectively induce apoptosis in glioblastoma and to increase the activity of TMZ, supporting the clinical evaluation of the PAC-1/TMZ combination in human glioblastoma patients.

## RESULTS

### Procaspase-3 is highly expressed in brain tumors and expression correlates with survival

**Figure 1:**
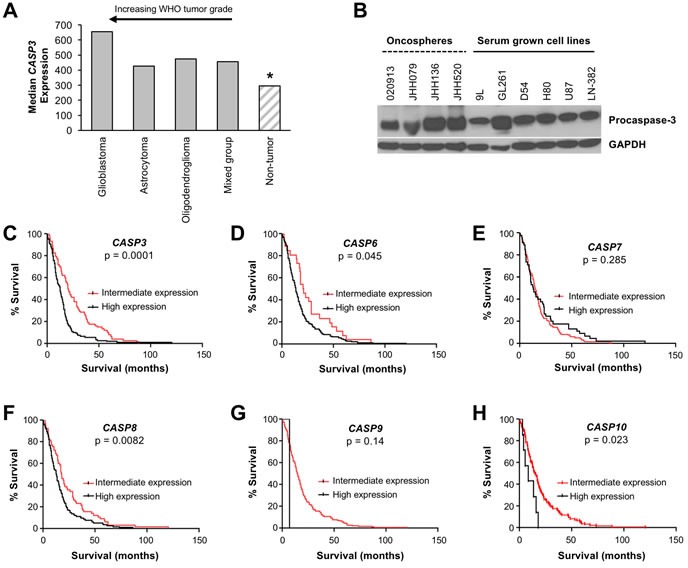
Procaspase-3 is expressed in brain tumors and its expression is a prognostic factor for survival of glioblastoma patients **A.** Analysis of *CASP3* expression in the NCI REMBRANDT database demonstrates that *CASP3* is more highly expressed in brain tumors compared to non-tumor tissues (*, *p* < 0.001 for pairwise comparison between non-tumor and glioblastomas or astrocytomas or oligodendrogliomas). Highest expression of *CASP3* was observed in glioblastoma, compared to lower expression in low-grade astrocytomas and oligodendrogliomas. **B.** Immunoblotting demonstrates the presence of robust procaspase-3 expression in glioblastoma cell lines. **C.**-**E.** Executioner caspases: *CASP3*, *CASP6*, and *CASP7*. **C.** Glioblastoma patients with high expression of *CASP3* survived significantly shorter (*n* = 107; median = 12.5 months) compared to those with intermediate *CASP3* levels (*n* = 74; median 20.3 months; *p* = 0.0001). **D.** High expression of the *CASP6* gene (*n* = 155) correlated with poorer prognosis when compared to intermediate expressors (*n* = 26) at a level of mild significance (*p* = 0.045). **E.** Expression of *CASP7* did not correlate with survival (*n* = 45 for high expression, *n* = 136 for intermediate expression). (**F**-**H**) Initiator caspases: *CASP8*, *CASP9*, and *CASP10*. **F.** Glioblastoma patients with high *CASP8* expression survived significantly shorter (*n* = 116; median = 13 months) compared to those with intermediate *CASP8* levels (*n* = 65; median 18 months; *p* = 0.0082). **G.** Expression of *CASP9* was primarily unchanged across the glioblastoma patients except one patient out of 181 exhibiting high caspase 9 expression and a shortened survival (*p* = 0.14). **H.** High expression of the *CASP10* gene (*n* = 7 for high expression, *n* = 137 for intermediate expression) correlated with poor prognosis at a level of mild significance (*p* = 0.023).

Given small data sets suggesting a potential increase in procaspase-3 levels in malignant brain tumors in comparison to benign tumors and normal brain tissue [14], a comprehensive retrospective survey was performed to determine if increased *CASP3* (encoding procaspase-3) expression affected prognosis in glioblastoma. Using the Repository for Molecular Brain Neoplasia Data (REMBRANDT) provided by the National Cancer Institute, expression of *CASP3* was analyzed in brain tumors and non-tumor controls. Analysis of REMBRANDT Affymetrix gene expression data demonstrates that elevated expression of *CASP3* correlates with tumor grade (Figure 1A). Non-tumor tissues had the lowest relative expression of *CASP3*, whereas glioblastomas had the highest expression of *CASP3* (*p*<0.001). Low-grade astrocytomas and oligodendrogliomas demonstrate intermediate transcript expression of *CASP3*. To extend upon *CASP3* gene expression data, protein levels of procaspase-3 in 10 glioblastoma cell lines (both serum-cultured and oncosphere lines) were analyzed by immunoblotting with an antibody recognizing procaspase-3. As a complement to traditional serum-grown cell lines, oncosphere cell lines were included as they better represent the genetic and histologic characteristics of a patients’ tumor than traditional serum-cultured adherent cell lines [30–32], and when used *in vivo* they retain many of the histopathological features characteristic of the cancer [31, 33]. All glioblastoma cell lines demonstrate robust expression of procaspase-3 (Figure 1B).

The REMBRANDT database was then used to analyze the survival of glioblastoma patients (n=181) based on expression of executioner caspase genes (*CASP3*, *CASP6* and *CASP7* for the caspase-3, -6, and -7 proteins) and initiator caspase genes (*CASP8*, *CASP9* and *CASP10* for the caspase-8, -9, and -10 proteins). Gene expression levels were determined using Affymetrix gene expression array and the genes were considered to be high expressing when the expression was greater than 2-fold higher compared to non-tumor samples. Similarly, genes were considered low-expressing when expression was less than 2-fold compared to non-tumor samples. Expression levels between 2-fold higher and 2-fold lower than non-tumor samples were considered to be intermediate and similar to non-tumor tissue. Consistent with IHC data [7, 13], high or intermediate expression was noted in all samples, with no low expressors (Figure 1C-1H). Kaplan-Meier analysis demonstrated a trend that high expression of caspase genes was usually associated with decreased survival in glioblastoma patients (Figure 1C-1H). Interestingly, the expression of *CASP9* was highly consistent across glioblastoma patients, with only one patient out of 181 exhibiting high *CASP9* expression (Figure 1G). The correlation between *CASP3* gene expression and prognosis was of greater significance than the correlation of any other caspase gene and prognosis (*p*=0.0001; Figure 1C). Glioblastoma patients with high expression of *CASP3* had significantly reduced survival compared to patients who demonstrated intermediate expression, suggesting that a compound directly activating procaspase-3 could have selective antitumor activity and be most beneficial in the patients with the poorest survival. A small panel of other proteins involved in the intrinsic and extrinsic apoptotic pathways were also evaluated (Supplementary Figure 1), but their expression did not reach or surpass the significance of *CASP3* expression.

### PAC-1 is cytotoxic to glioma cells in culture as a single agent and enhances the activity of TMZ

**Figure 2:**
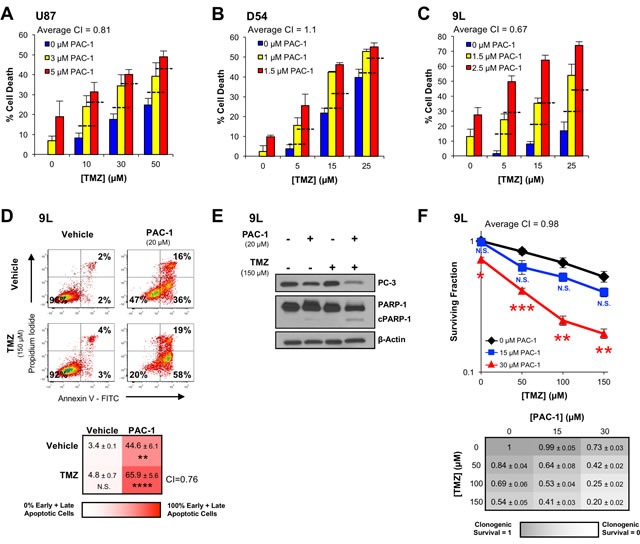
PAC-1 and TMZ induce synergistic cell death in glioblastoma cells **A.**-**C.** Glioblastoma cells (**A**: U87, human; **B**: D54, human; **C**: 9L, rat) were treated for 72 h with combinations of PAC-1 and TMZ (assessed by Alamar Blue). The dashed horizontal lines represent the level of cell death expected from an additive effect of compounds. The average Combination Index (CI) value across the combinations is indicated for each cell line. CI values < 1 are synergistic, with lower values indicating higher levels of synergy. CI values = 1 indicate an additive effect. **D.** The ability of PAC-1 and TMZ to induce apoptosis in 9L cells was assessed by flow cytometry with Annexin V-FITC and propidium iodide staining after a 48 h treatment. A substantial apoptotic population (FITC + / PI -) was observed with the combination treatment. Flow plots of representative data are shown. Mean of three independent experiments and standard error are indicated for each combination in the grid below. Statistical comparison to vehicle treated cells ** = *p* < 0.005, **** = *p* < 0.0005*.* **E.** 9L cells treated with PAC-1 and TMZ were analyzed *via* Western blot for the presence of markers of apoptosis, procaspase-3 activation and PARP-1 cleavage, after a 48 h treatment with compounds. **F.** Combination treatments of PAC-1 and TMZ were assessed for their ability to impact the clonogenic potential of 9L cells. Cells were treated for 12 h and clonogenic growth was assessed after 7 days. Mean and standard error are indicated for each combination in the table below. Statistical comparison to TMZ-only clonogenic survival * = *p* < 0.01, ** = *p* < 0.005, *** = *p* < 0.001. The average Combination Index (CI) value across the combinations is indicated in the grid below the graph. *n* = 3 biologic replicates for all experiments. N.S. indicates a not significant difference.

The ability of PAC-1 to induce death in a panel of glioma cell lines, consisting of both serum grown and oncosphere lines, was analyzed. PAC-1 demonstrated a median IC_50_ value of ∼5 μM in serum grown lines, and ∼18 μM against oncosphere cells (Supplementary Table 1). After establishing that PAC-1 was cytotoxic as a single agent in brain cancer cell lines, combination studies with the standard-of-care for the treatment of glioblastoma, TMZ, were conducted. PAC-1 was evaluated in combination with TMZ in a small panel of cancer cell lines in culture, including those of human (U87 and D54) and rat (9L) origin. While D54 and 9L are malignant glioma lines, the origin of U87 is in doubt, but likely resulted from contamination from a different glioblastoma line than the original patient cultured [34]. In these experiments additive to synergistic (supra-additive) responses were observed (Figure 2A-2C), as defined by Combination Index analysis [35]. Additional characterization of cell culture anticancer activity was measured in the 9L cells, as a prelude to orthotopic *in vivo* models. Treatment of 9L cells with a combination of PAC-1 and TMZ induced synergistic apoptosis, above the additive effects of each single agent, as determined by Annexin-V-FITC and propidium iodide staining (Figure 2D). Furthermore, Western blot analysis of the combination treated cells demonstrated the presence of substantial cleavage of procaspase-3 and PARP-1, characteristic markers of apoptosis (Figure 2E). The PAC-1 and TMZ combination was also assessed for the ability to alter the clonogenic potential of 9L cells. In this experiment a significant decrease was seen in the clonogenic survival of the combination-treated cells relative to the single-agent effect (Figure 2F).

### PAC-1 does not induce off-target activation of procaspase-3 in neurons and glial cells

As a prelude to evaluating PAC-1 efficacy in murine models of glioblastoma, the effect of PAC-1 treatment on neurons, ependymal cells, and supporting glial cells were assessed in normal murine brain tissues. For this study, healthy male and female C57BL/6 mice were administered PAC-1 orally at a dosage of 50 mg/kg (as an aqueous suspension) daily for 14 consecutive days, which mirrored PAC-1 exposures planned for anticancer efficacy studies. Mice maintained normal behavior and body weights throughout the duration of the study, and no clinically observable signs of neuroexcitation were detected.

### PAC-1 is efficacious in a syngeneic orthotopic glioblastoma rodent model

**Figure 3:**
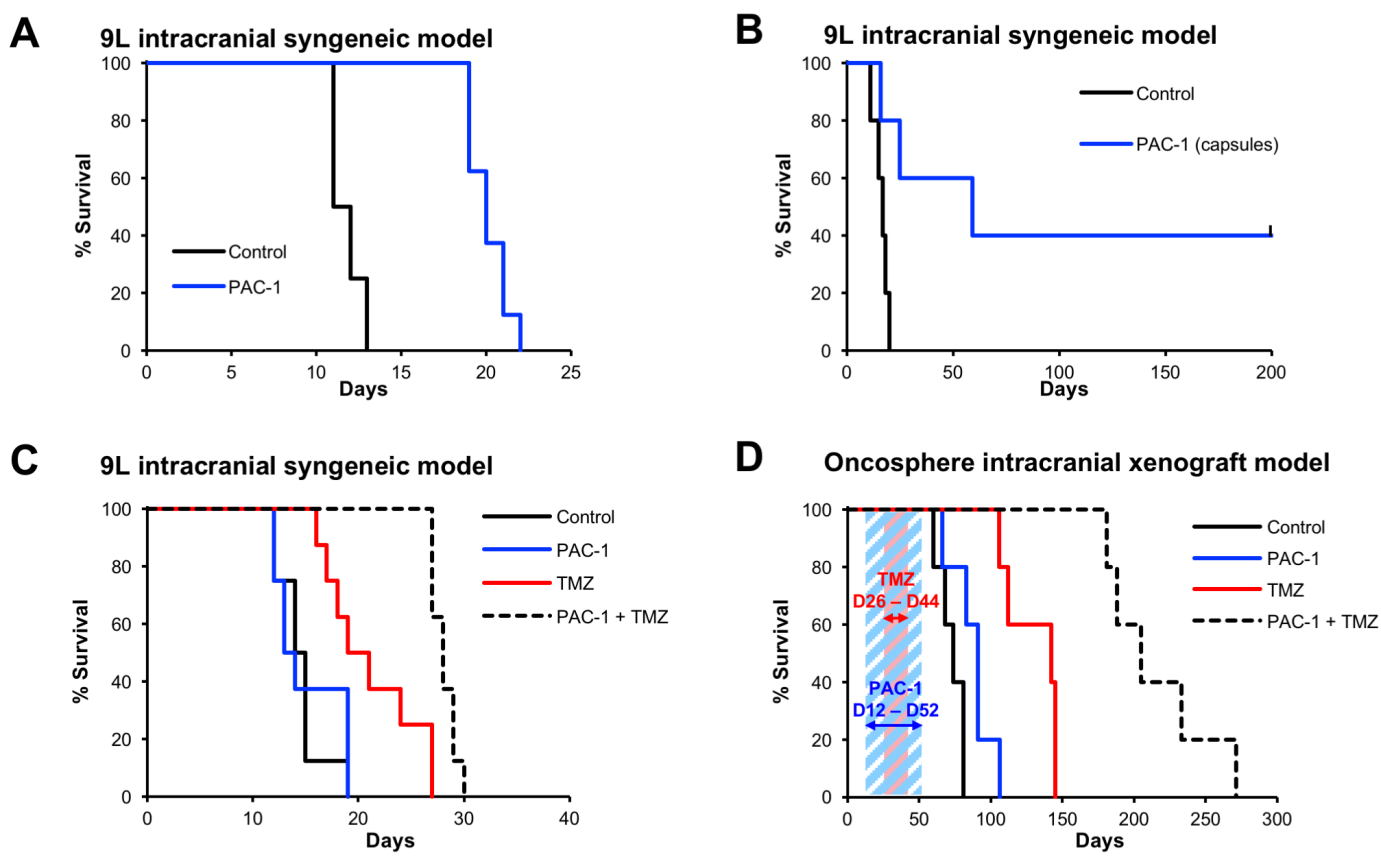
Oral PAC-1 is efficacious as a single agent in an intracranial model of glioblastoma and synergistically enhances the efficacy of TMZ in intracranial models of glioblastoma **A.** PAC-1 improved survival of 9L rats by 73% (*p* < 0.001). Rats received 10 oral doses (50 mg/kg, in an aqueous oral suspension, days 5-9 and 12-16). The median survival of control animals was 11.5 days, compared to 20 days in PAC-1 treated animals. 8 rats per group. **B.** PAC-1, administered in a gelatin capsules, improved survival of 9L rats by 350% (*p* = 0.02), from 17 days to 59 days. Rats received 16 oral doses (50 mg/kg, solid packed into size 9 capsules, days 3-18). 5 rats per group. **C.** Combinations of PAC-1 and TMZ synergistically extend survival in intracranial 9L rat glioblastoma. PAC-1 (50 mg/kg, administered in an aqueous oral suspension, days 1-5), did not improve survival as a single agent (median survival 13.5 days compared to 14.5 days in the control group), but did extend survival when used in combination with TMZ (50 mg/kg, oral, days 6-10; TMZ alone median survival: 20 days, PAC-1 + TMZ median survival: 28 days). The combination of PAC-1 + TMZ extended survival to a statistically significant extent compared to control rats (*p* < 0.0001) and TMZ as a single agent (*p* = 0.007). 8 rats per group. **D.** Combinations of PAC-1 and TMZ synergistically extend survival in intracranial oncosphere-derived glioblastoma (cell line 020913) in NOD SCID mice. PAC-1 (50 mg/kg, oral, 5 days a week from day 12-52, shown with blue diagonal shading) improved survival from 74 days (control) to 91 days (*p* = 0.034). TMZ alone (50 mg/kg, oral, 5 days a week from day 26-44, shown with red diagonal shading) improved survival to 142 days. The combination of PAC-1 and TMZ was most effective, extending the median survival to 205 days (*p* = 0.0027 compared to TMZ, *p* < 0.0001 compared to control). 5 mice per group.

Cell culture experiments demonstrated that PAC-1 was efficacious as a single agent across a panel of brain cancer cell lines, including 9L rat glioma cells. Preliminary investigations of the efficacy of PAC-1 *in vivo* were initiated in the highly aggressive syngeneic 9L rat glioma model, with 9L cells intracranially implanted. In this experiment, rats received PAC-1 (50 mg/kg, oral, as an aqueous suspension) from day 5 to day 9, and day 12 to day 16, for a total of 10 doses. The median survival in control animals (n=8) was 11.5 days compared to 20 days in PAC-1 treated rats (n=8; *p* < 0.001); the ten doses of PAC-1 provided a 73% increase in median survival suggesting a substantial anti-glioma activity for PAC-1 as a single agent (Figure 3A).

The efficacy of PAC-1 was then evaluated when administered as a solid in gelatin capsules, a formulation with greater translational relevance. Rats implanted with intracranial 9L glioma cells were administered PAC-1 (50 mg/kg) in capsules once-a-day for 16 consecutive days beginning on day 3 post-tumor implantation. Rats treated with PAC-1 in capsules showed significantly improved survival; the median survival for rats treated with PAC-1 capsules (n = 5, median survival = 59 days) was 350% longer than for the untreated control rats (n = 5, median survival = 17 days, *p* = 0.02; Figure 3B), and 40% of the animals survived until the end of the experiment (day 200).

### PAC-1 synergizes with TMZ in orthotopic glioblastoma rodent models

The ability of PAC-1 to synergize with TMZ *in vivo* was first evaluated in the intracranial 9L syngeneic model of glioma. For these experiments the PAC-1 treatments were limited to five times total, thereby minimizing its single agent activity relative to the experiments in Figures 3A&3B, where animals were dosed with PAC-1 10 or 16 times. Rats (n = 8 per group) were treated with water (vehicle control), PAC-1 as a single agent (50 mg/kg, oral, as an aqueous suspension, days 1-5), TMZ as a single agent (50 mg/kg, oral, days 6-10), or the combination of PAC-1 + TMZ. As shown in Figure 3C, minimal dosing of PAC-1 did not alter animal survival (median survival of PAC-1 alone: 13.5 days, median survival of vehicle control rats: 14.5 days, *p* = 0.75). TMZ improved the median survival to 20 days, and was significantly longer than control rats, *p* = 0.0018. Treatment with PAC-1 in combination with TMZ significantly improved the median survival (to 28 days) in comparison to the control group (*p* < 0.0001) as well as TMZ only group (*p* = 0.007), demonstrating synergy between PAC-1 and TMZ *in vivo*.

PAC-1 and TMZ were evaluated in a second and more clinically relevant model, a murine intracranial xenograft model with oncospheres in NOD SCID mice. The cell line 020913 grows as oncospheres and forms gliomas that retain the original histopathological and genetic features of the original tumor [36]. Groups of mice (n = 5 per group) were treated with vehicle, PAC-1 as a single agent (50 mg/kg, oral, five days a week for 6 weeks beginning on day 12), TMZ as a single agent (50 mg/kg, oral, five days a week for 3 weeks beginning day 26), or the oral combination of PAC-1 + TMZ (Figure 3D). Importantly, the trends seen in previous models were also observed in this oncosphere model. Single agent PAC-1 extended survival from 74 days (vehicle treated mice) to 91 days (*p*=0.034), and TMZ alone extended survival to 142 days (*p*=0.0027). Use of PAC-1 in combination with TMZ substantially improved the median survival to 205 days (*p*<0.0001 compared to vehicle, *p*=0.0027 compared to TMZ alone).

### Addition of PAC-1 to TMZ and ionizing radiation is feasible and associated with cytoreductive activities in canine cancer patients

**Figure 4:**
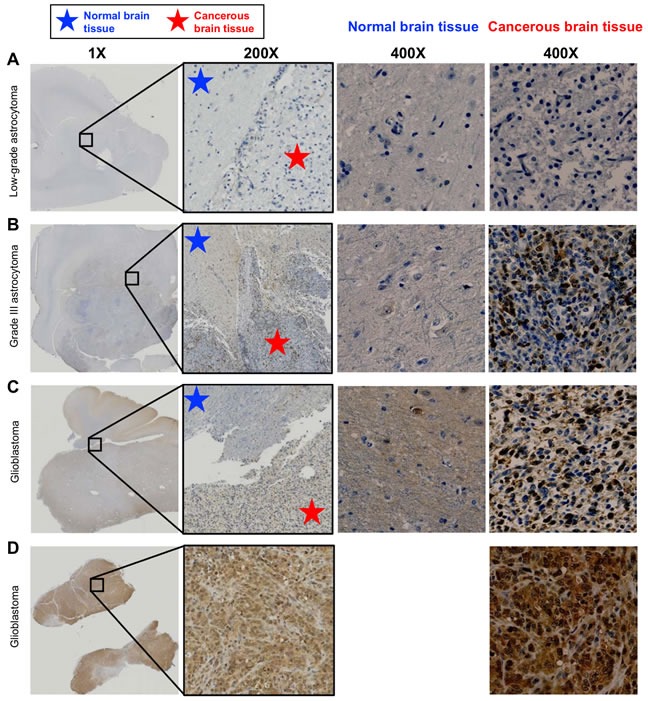
Procaspase-3 immunohistochemical staining of spontaneous canine astrocytomas A portion of the low power image (0.5X) on the far left is enlarged to 200x in the image at right. The blue and red stars show the approximate areas of tumor and normal tissue; some PC-3 positive tumor cells can be observed infiltrating between the tumor mass and normal tissues in images B and C. **A.** A low grade astrocytoma tumor from a necropsy specimen did not stain positively for procaspase-3. **B.** Grade III astrocytoma, necropsy specimen. ∼30-40% of cells in the tumor tissue stained with moderate to strong cytoplasmic PC-3 staining intensity; both the cellularity and staining intensity are increased relative to normal brain tissue. **C.** GBM, necropsy specimen. Over 90% of cells have moderate cytoplasmic PC-3 staining intensity in this tumor; again, both the cellularity and staining intensity are increased relative to normal brain tissue. **D.** GBM biopsy tissue. > 90% of cells contained moderate to strong cytoplasmic PC-3 staining intensity. This staining intensity is markedly increased as compared to the normal tissues observed in images A, B and C. No normal tissue was available in this sample.

Canine cancer patients offer the opportunity to evaluate the feasibility of combining PAC-1 with conventional cytoreductive treatment strategies in large mammalian patients. Importantly, human and canine astrocytomas and gliomas present with similar morphologies, pathologies and molecular abnormalities [37–39]. The pharmacokinetics and efficacy of PAC-1/derivatives has been well studied in pet dogs with cancer, including those with lymphoma and metastatic osteosarcoma [26, 40, 41]. Prior to initiating a feasibility study of PAC-1 in pet dogs with brain cancer, the suitability of PAC-1 targeted therapy for canine glioma was determined using a small cohort (n = 4) of archived paraffin-embedded tumor samples. Spontaneously arising canine astrocytomas low-grade (n = 1), grade III (n = 1) and glioblastomas (n = 2) were assessed for immunohistochemical procaspase-3 staining intensity. A general trend of increasing procaspase-3 staining intensity with increased malignancy was present (Figure 4), with procaspase-3 staining being notably less intense in normal brain tissue and not identifiable in a low grade astrocytoma. This contrasted with a Grade III astrocytoma tissue sample displaying 30-40% cytoplasmic staining for procaspase-3, and glioblastoma samples showing 90% positive staining. Importantly, in three of the canine samples, normal brain tissue was directly adjacent to neoplastic tissue, and in these cases the tumor tissue contained a higher degree of intracytoplasmic procaspase-3 staining intensity as compared to the normal surrounding tissue (Figure 4).

**Figure 5:**
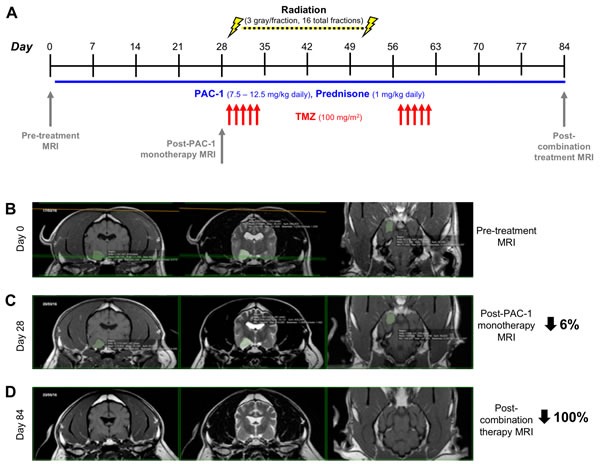
Evaluation of combining oral PAC-1 with TMZ and definitive ionizing radiation therapy in pet dogs with non-resectable gliomas **A.** Schematic of treatment schedule. PAC-1 was administered orally from days 1-84 (as shown by the blue horizontal line). TMZ was administered orally days 29-33 and 57-61 (indicated by red arrows). **B.**-**D.** MRI of Patient 1's glioma on Day 0 (B, pre-treatment), Day 28 (C, post PAC-1 monotherapy), and Day 84 (D, post combination therapy). Tumors have been false-colored green. Change in tumor size from Day 0 is denoted on far right side of images. Quantification in Table 1.

With the goals of evaluating the feasibility of PAC-1 in combination with TMZ and concurrent ionizing radiation, as well as assessing *in vivo* activity, the combination was evaluated in three pet dogs diagnosed via MRI with naturally-occurring intra-axial, non-resectable gliomas (Patients 1-3). As displayed in Figure 5A, canine patients were treated with oral PAC-1 (7.5 – 12.5 mg/kg) for 84 consecutive days. Canine patients also received 100 mg/m^2^ oral TMZ on days 29-33 and 57-61. Ionizing radiation (3 gray/fraction) was administered between days 29-52 on a Monday-Friday schedule for 16 total fractions (48 total gray). 3D conformal radiation plans were developed with Eclipse 11.0 (Varian Medical Systems) and administered via 6MV photons from a Varian 21EX linear accelerator. Patients received daily prednisone (1 mg/kg) throughout the study. Pre-treatment MRI was performed prior to initiation of investigational therapy with oral PAC-1 as a monotherapy, a second MRI was performed to assess for single agent PAC-1 activity, and a third MRI on day 84 was performed, upon the completion of multimodality therapy exposure. Blood was collected for hematologic and non-hematologic assessment at pre-treatment, on Days 29, 57, and 84. Importantly, all patients completed the treatment protocol without severe toxicities and no mortalities were observed over the course of the study, suggesting that addition of oral PAC-1 to the protocol is highly feasible. The only notable toxicity observed was in one dog that developed cerebellar related tremors and ataxia after starting PAC-1 that resolved with dose reduction. Two of three patients exhibited minimal tumor decreases following PAC-1 monotherapy (6% and 5% for Patients 1 and 2, respectively, Table 1). Further, all three patients exhibited objective response to the complete therapy protocol, with Patient 1 exhibiting a complete response (100%) and eradication of tumor (MRI images of Patient 1 shown in Figure 5B-5D), and Patients 2 and 3 exhibiting strong partial responses, with 43% and 60% reductions in tumor burden, respectively (MRI images for Patients 2 and 3 shown in Supplementary Figure 2, results summarized in Table 1).

**Table 1:** Canine glioma patients treated with PAC-1, TMZ and ionizing radiationa

					Change in Tumor Burden Following Treatment	
Patient	Breed	Age	Sex	PAC-1 dosage (mg/kg)	PAC-1 monotherapy	Combination therapy
1	Labrador retriever	7	FS^a^	12.5^b^	↓ 6%	↓ 100%
2	Dachshund	8	FS	10.0	↓ 5%	↓ 43%
3	Boxer	5	FS	7.5	↑ 19%	↓ 60%

^a^Abbreviations: FS; spayed female. ^b^PAC-1 dosage was 12.5 mg/kg for days 1-56, then reduced to 8.0 mg/kg for days 57-84.

## DISCUSSION

While survival has steadily increased for several common cancers, therapy for glioblastoma still only results in a median patient survival of about 15 months [4, 5]. No new small molecules have been approved for the treatment of glioblastoma following the 2005 approval of TMZ. This lack of novel chemotherapeutics exists even as knowledge of the molecular aberrations found in glioblastomas expands rapidly. Glioblastoma was one of the first cancers profiled by the National Institutes of Health's Cancer Genome Atlas [42], and these efforts identified numerous promising targets including EGFR, VEGF, PDGFRs and PKC -- all targets that were observed to be frequently overexpressed in glioblastomas and for which overexpression frequently correlated inversely with prognosis. Furthermore, small molecules targeting these proteins have been tested for the treatment of diverse and challenging solid tumors (such as non-small cell lung cancer), have demonstrated survival increases, and have been FDA approved [43]. Unfortunately, no clinical trials of molecularly targeted small molecules in glioblastoma to date have yielded a survival benefit for patients. Thus, there exists a significant challenge in translating the evermore sophisticated knowledge of glioblastoma molecular aberrations into effective and durable responses in patients.

Numerous factors drive the lethality and resistance to chemotherapies of glioblastoma, but the disappointing lack of activity of novel molecularly targeted agents can be largely attributed to two mechanisms: lack of complete target suppression, and engagement of feedback and overlap between targeted signaling pathways. The requirement for complete inhibition of a target protein can drive resistance in many cancers, but glioblastomas possess the added challenge of achieving therapeutically sufficient drug levels at tumor sites within the brain. In this regard, a BBB-penetrant small molecule *activator*, such as PAC-1, may have advantages. While near-quantitative inhibition is often needed for an effect of a small molecule enzyme inhibitor, the activation of a low percentage of cellular procaspase-3 by PAC-1 can set off a feed-forward cycle, with the caspase-3 generated activating additional procaspase-3 [22, 26].

PAC-1 is a blood-brain barrier penetrant small molecule, capable of bypassing intrinsic anti-apoptotic mechanisms and initiating apoptosis in cancer cells and therefore represents an opportunity for intracranial tumors, particularly for those such as glioblastoma where procaspase-3 is strongly expressed. PAC-1 distributes between the brain and the blood with a 30:70 ratio [23], and the CSF:blood ratio of TMZ has been reported as 17:83 in humans [44], suggesting the potential of these two proapoptotic agents to act synergistically *in vivo*. Indeed, as described herein the combination of PAC-1 and TMZ was synergistic in orthotopic rodent glioma models. More significantly, the combination of PAC-1, TMZ, and radiation was well-tolerated in pet dogs with glioma, and a clinical response was observed in all three of these canine cancer patients.

There are some caveats when interpreting the canine data. First, the number of subjects is necessarily small, not allowing for a full randomized trial comparing standard of care to standard of care plus PAC-1. Secondly, the dosing and duration of TMZ plus radiation is adapted to canine therapy and differs from the human regime with regards to dose intensity. Finally, for intracranial masses, while consistent for intra-axial glial tumors based upon MRI findings, the specific tumor histology was not confirmed at diagnosis for the small cohort of pet dogs enrolled.

The BBB presents a significant challenge for treatment of intracranial tumors, thus it is critical that novel experimental therapeutics are assessed in *in vivo* settings that closely mimic the human disease. Although rodent models of cancer have been exceptionally useful in rapidly and systematically evaluating potential cancer treatment strategies in a controlled manner, scores of drugs show efficacy in these models, but this activity very often fails to translate to human cancer patients; indeed, only ∼1 in 20 oncology candidates that start a Phase I trial ultimately becomes an approved drug [45]. Most rodent tumor models rely upon rapidly growing, homogeneous tumors that frequently demonstrate limited invasion or metastases. In addition, rodents have a highly abbreviated life span compared to humans. Thus, the evaluation of anticancer strategies in companion animals (pets) with spontaneous cancers can complement induced murine models [46, 47].

Canine patients with naturally occurring brain tumors represent an emerging resource to test the feasibility, tolerability and activity of anticancer strategies [48]. Spontaneous canine cancers develop with tumor heterogeneity more representative of the human disease than murine models of cancer [49, 50]. Canine patients are closer in weight, metabolism, and pharmacokinetics to the analogous human physiology than are rodents. Canine gliomas present with similarities to their human counterparts, including relative age of onset, phenotypes, chromosome copy number aberrations, and gene expression profiles [51, 52]. Gliomas, like many canine cancers, display a breed-specific enrichment, in this case in short-nosed dog breeds (brachycephalic) [51].

Due to the expense of imaging, radiation therapy, and chemotherapy treatment with TMZ, limited literature precedent for the expected outcome of glioblastoma therapy in canine patients exists, although anecdotally, complete regressions are rarely observed in canine brain tumors. Thus, the demonstrated feasibility of combining PAC-1 with conventional anti-glioma therapies such as TMZ and ionizing radiation in pet dogs with spontaneous glioma provides highly valuable translational data that can be leveraged to expedite and guide the implementation of similar therapeutic strategies for human glioblastoma patients. PAC-1 is currently being evaluated in human cancer patients in a Phase I clinical trial (NCT02355535). Based upon its ability to penetrate the BBB, to bypass anti-apoptotic mechanism to induce cell death, its activating mode-of-action, and the over-expression of procaspase-3 glioblastoma, PAC-1 has the potential as an orally delivered small molecule with a capacity for both single agent anti-glioblastoma activity, as well as the ability to synergize with TMZ to increase activity and achieve more durable responses in patients.

## MATERIALS AND METHODS

### Immunohistochemistry, cell viability, apoptosis, and clonogenic survival

Glioblastoma oncosphere cells were cultured in serum free medium cultured in DMEM supplemented with 10% FBS and 1X Penicillin/Streptomycin. All cells were maintained at 37°C in 5% CO_2_ at low passage.

To determine basal procaspase-3 expression in glioma cell lines, protein lysates were prepared using RIPA buffer, normalized by a bicinchoninic acid assay, denatured, separated on a denaturing gradient gel and transferred to a PVDF membrane. Procaspase-3 protein levels were determined (Cell Signaling #9665), and GAPDH was used as a loading control.

IC_50_ values for PAC-1 and TMZ were determined following a 72 hour incubation with a range of concentrations by Alamar blue (DMSO vehicle control was normalized to 1%). PAC-1 and TMZ were assessed in combination in U87, D54 and 9L cells (1,875 per well) treated for 72 h with a range of TMZ concentrations in the presence or absence of three concentrations of PAC-1. Cell number and viability were assessed by Alamar Blue.

The ability of PAC-1 and TMZ combinations to induce apoptosis was assessed by Annexin V /propidium iodide staining and western blot analysis of PARP-1 cleavage and cleavage of procaspase-3. 50,000 9L cells were plated in 6-well plates and treated with combinations of PAC-1 (20 μM) and TMZ (150 μM) for 48 hours. At the conclusion of treatment the entire contents of each well was pelleted and assessed by either flow cytometry or immunohistochemistry. Pellets for flow cytometry were suspended in 450 μL of Annexin V-FITC binding buffer (10 mM HEPES, 140 mM NaCl, 2.5 mM CaCl_2_, 1% BSA, pH 7.4), premixed with Annexin V-FITC and propidium iodide dyes. Staining was assessed by flow cytometry (10,000 events recorded per sample). Pellets for immunohistochemistry were prepared as described above and analyzed for procaspase-3 (Cell Signaling #9662) and PARP-1 (Cell Signaling #9542). β-actin (Cell Signaling #4970) served as a loading control.

Alterations of clonogenic survival was assessed in 9L cells. 250 cells were allowed to adhere for 48 hours before treatment with combinations of PAC-1 and TMZ (12 hours). Media was replaced, plates sealed gas permeable membranes and colonies were allowed to form (7 days). At the conclusion of colony growth, colonies were washed with PBS and stained with 0.05% crystal violet in 6% glutaraldehyde for 30 minutes. Colonies were defined as >50 cells, and the cologenic survival was determined as the number of colonies formed after treatment divided by the number of cells seeds multiplied by the plating efficiency; incongruence with established methods to assess clonogenic survival.

### Analysis of glioblastoma patient survival by gene expression

The REMBRANDT database from the NCI was used to analyze the survival of glioblastoma patients based on expression of key genes involved in extrinsic and intrinsic apoptosis pathway. Gene expression levels were determined using Affymetrix gene expression array and the genes were considered to be high expressing when the expression was > 2-fold compared to non-tumor expression. Similarly, the genes were considered to be low-expressing when their expression was < 2-fold compared to non-tumor expression. Expression between those cut-offs was considered as intermediate and representative of not distinctly different compared to non-tumor expression.

### Assessment of procaspase-3 immunostaining in normal and malignant canine brain tissue

The University of Illinois Veterinary Diagnostic Laboratory database was reviewed for cases of canine astrocytoma, and identified tissues were recut for procaspase-3 immunohistochemistry. Processed slides were deparaffinized in xylene and rehydrated in alcohol. Endogenous peroxidase activity was blocked with Biocare #PX968 Peroxidazed 1 at room temperature for 5 minutes, rinsed with TBS wash buffer, and then incubated for 10 minutes at room temperature with Biocare #BP974 Background Punisher. Slides were incubated with procaspase-3 antibody (Abcam #ab32150) for 39 minutes at a dilution of 1:3000, washed, and then incubated with Rabbit-on-Canine HRP-Polymer (Biocare #RC542) for 30 minutes. Slides were washed in TBS wash buffer. The reaction was developed using DAB substrate for 5 minutes and the slides were then counterstained with Mayer's hematoxylin. Canine lymph nodes served as negative and positive controls.

### Intracranial orthotopic rodent models

9L glioma, syngeneic in F344 rats

9L tumors (2 mm^3^) were passaged in the flanks of F344 carrier rats (female, 150–200 g). For intracranial implantation, the 9L tumor was surgically excised, cut into 1 mm^3^ pieces, and placed in sterile 0.9% saline on ice until implantation. F344 female rats (weighing 150-200 g) were anesthetized, their heads shaved and prepared with povidone-iodine, and a midline scalp incision was made. With an electric drill, a burr hole (3 mm) was drilled 3 mm lateral to the sagittal suture and 5 mm posterior to the coronal suture. A small amount of cortex and white matter was removed with gentle suction. The 1 mm^3^ piece of 9L tumor was placed implanted and the skin was closed with surgical sutures. Rats were randomized prior to receiving treatments.

To determine the efficacy of PAC-1 (suspension), rats (n=8 per group) received 10 oral doses (50 mg/kg) on days 5-9 and 12-16, and survival was compared to untreated animals. To determine the efficacy of PAC-1 in capsules, rats (n=5 per group) were treated with PAC-1 in capsules (50 mg/kg, in size 9 Torpac capsules, using a dosing syringe) for 16 consecutive days starting day 3, and survival was compared to untreated animals.

To determine the efficacy of PAC-1 in combination with TMZ, rats were implanted with 9L tumors as previously, and randomly divided into four treatment groups (n=8 per group). Rats treated with PAC-1 (alone, or in combination with TMZ), received PAC-1 (50 mg/kg, oral suspension, days 1-5). Mice treated with TMZ (alone, or in combination with PAC-1) received TMZ (50 mg/kg, oral, days 6-10).

### Glioblastoma oncospheres (020913), xenograft in NOD SCID mice

Twenty male NOD SCID mice were anesthetized with a mixture of ketamine and xylazine and a small incision in the skin over the cranium was made. A hole was made 1 mm lateral of midline and 1 mm lateral of Bregma over the parietal lobe using a surgical drill. The animal was placed in a stereotactic frame and 360,000 020913 cells were implanted at a depth of 2.5 mm using a Hamilton syringe. The incision was closed with surgical staples. Mice were randomly divided into four treatment groups (n=5 per group). Mice treated with PAC-1 (alone, or in combination with TMZ), received PAC-1 (50 mg/kg, oral) 5 days a week from day 12-52. Mice treated with TMZ (alone, or in combination with PAC-1) received TMZ (50 mg/kg, oral) 5 days a week from day 26-44.

### Feasibility of combining PAC-1 with oral temozolomide and ionizing radiation in pet dogs diagnosed with intra-axial glial tumors

Pet dogs with spontaneously-arising intra-axial glial tumors based upon characteristic MRI findings were recruited for voluntary participation in a feasibility trial evaluating the safety and activity of combining oral PAC-1 with temozolomide and ionizing radiation. Pet owners were educated on the investigational nature of PAC-1, and provided expert guidance regarding all medically-indicated treatment options that could be pursued for the management of their pet dog prior to study enrollment. Upon informed client consent, pet dogs had standardized diagnostics performed including neurologic examination, screening blood work, and MRI, and then treated with a standardized protocol inclusive of PAC-1, TMZ, and ionizing radiation therapy lasting over a time span of 84 days. The instituted combination treatment protocol of PAC-1, TMZ, and ionizing radiation in pet dogs, while analogous to standard-of-care therapy in people diagnosed with GBM, was less dose-intense with regards to TMZ dosage and cumulative ionizing radiation dose delivered, as compared to conventional human protocols.

### Serial MRI assessment protocol

To document cytoreductive activities of PAC-1 alone or in combination with oral TMZ and ionizing radiation therapy, pet dogs had a total of 3 serial MRI scans at Day 0 (pre-treatment), Day 28 (PAC-1 monotherapy), and Day 84 (PAC-1 combined with TMZ and ionizing radiation). A semi-automated 2-dimensional segmentation process using OsiriX (OsiriX 64-bit, version 8.0.2, Pixmeo, Switzerland) was employed to grow areas of interest based on voxel signal intensity thresholds which delineated tumor size. The baseline threshold was set on Day 0 (pre-treatment) MRI scans and included the whole tumor and no adjacent structures of similar signal intensity. This threshold was then applied to all subsequent MRI scans (days 28 and 84). A combination of T2w, T1w and T1w with contrast images were used to make direct comparisons in tumor size change serially across MRI scans. The resulting semi-automated segmentation allowed regions of interest to be consistently and uniformly calculated within individual patients (dimensional planar as mm^2^).

### Statistical analysis

Survival curves were analyzed with Graphpad Prism 5.0 and log rank test was used to determine the significance. A *p* value of less than 0.05 was regarded as statistically significant.

## SUPPLEMENTARY FIGURES AND TABLE


