# Supplementary material for: Synergistic and targeted therapy with a procaspase-3 activator and temozolomide extends survival in glioma rodent models and is feasible for the treatment of canine malignant glioma patients
Source: Oncotarget. 2017 Jul 7;8(46):80124–38. doi: 10.18632/oncotarget.19085 (PMC5655184; doi:10.18632/oncotarget.19085)
Supplement: Supplementary file 1 [file oncotarget-08-80124-s001.pdf]

Synergistic and targeted therapy with a procaspase-3 activator and temozolomide extends survival in glioma rodent models and is feasible for the treatment of canine malignant glioma patients

Supplementary Material

MATERIALS AND METHODS

Compounds

TMZ for animal studies was obtained from Johns Hopkins Pharmacy. TMZ for cell culture studies was purchased from Selleck. PAC-1 was synthesized as previously reported [1].

Cell culture

Glioblastoma oncosphere cells 020913 were a kind gift from Sara Piccirillo and Angelo Vescovi, Università degli Studi Bicocca-Millan, Italy. Other glioblastoma oncosphere cell lines JHH136, JHH 520 and JHH079 were generated from patients tissues obtained from Johns Hopkins Hospital using an IRB approved protocol. All the glioblastoma oncosphere cells were cultured in serum free medium supplemented with EGF (20 ng/ml) and FGF (10 ng/ml) (Peprotech). Glioblastoma cell line LN-382 was gifted by Dr. Erwin Van Meier at Emory University and 9L glioma cells were a generous gift from Marvin Barker, MD, (University of California, San Francisco, Brain Tumor Research Center, San Francisco, CA). The serum-grown/adherent cell lines were grown in DMEM supplemented with 10% FBS and 1X Penicillin/Streptomycin (Life Technologies, Grand Island, NY). All the cells were maintained at 37˚ C in 5% CO_2 at low passage.

Animal study experimental design

All animal experiments were conducted under an approved protocol with either the Johns Hopkins Animal Care and Use Committee or the University of Illinois Institutional Animal Care and Use Committee. Animals were randomized prior to initiation of treatment.

Assessment of cleaved caspase-3 in normal murine brain tissue

Mice were treated for 14 days with (PAC-1, oral, 50 mg/kg), sacrificed and cleaved caspase-3 immunohistochemistry was performed on brain tissues to characterize potential off-target blood-brain barrier penetrant side effects, specifically apoptosis induction of normal bystander neurons or glial cells. Processed brain slides were deparaffinized in xylene and rehydrated in alcohol. Endogenous peroxidase activity was blocked with 3% hydrogen peroxide in methanol for 15 minutes, followed by 2 rinses in IHC wash buffer (Biogenex, San Ramon CA) for 5 minutes. Slides were placed in citrate buffer, pH 6, and put into the Decloaker (Biocare Medical) with deionized water. Slides were cooled in citrate buffer for 2 minutes, and then placed in wash buffer. Using an automatic immunostainer (i6000, Biogenex), non-specific staining was minimized by blocking for 15 minutes with avidin and biotin. Slides were then incubated with normal goat serum for 20 minutes at room temperature, then incubated with cleaved caspase-3 primary antibody (Cell Signaling, #9661; 1:1250 dilution) at 4°C for 30 minutes, washed, and then incubated with Supersensitive rabbit link (BioGenex) for 20 minutes at room temperature. Slides were again washed and incubated with Supersensitive HRP Reagent (BioGenex) for 20 minutes at 22-25°C. The reaction was developed using DAB substrate for 5 minutes and the slides were then counterstained with Mayer's hematoxylin. A murine T-cell lymphoma (EL4) cell pellet treated with staurosporine served as a positive control.

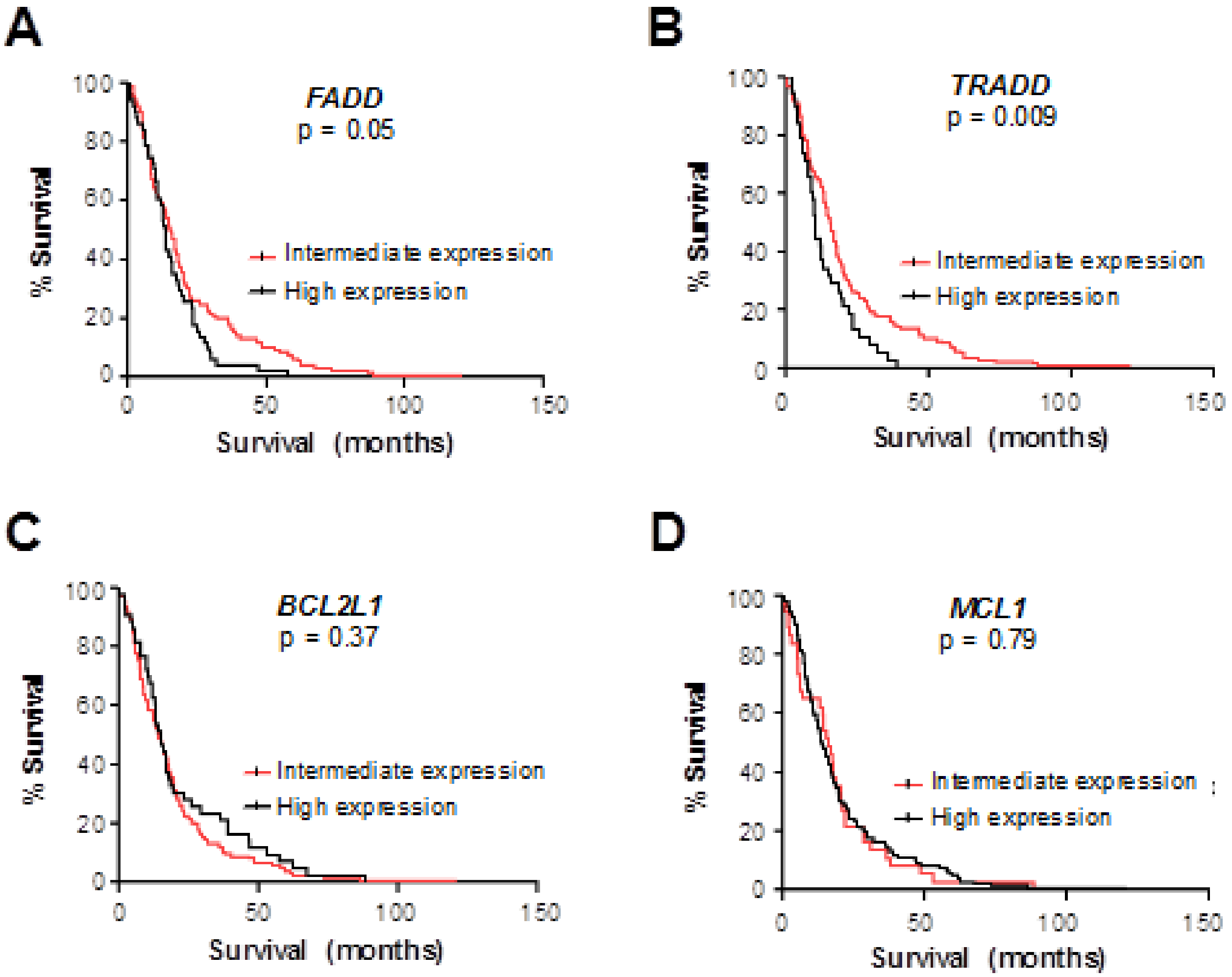

Supplementary Figure 1: Kaplan Meier analysis of selected apoptosis pathway genes in glioblastoma patients. Analysis for apoptosis pathway genes are correlated with poor survival to a mild significance, whereas intrinsic apoptosis pathway genes (*BCL2L1*, *MCL1*) expression does not predict survival using a Log rank test. **(A)** Glioblastoma patients with high *FADD* tend to survive shorter (n=51; median=13.3 months) compared to those with intermediate *FADD* levels (n=130; median 15.4 months; *p*=0.052). **(B)** Glioblastoma patients with high *TRADD* survived significantly shorter (n=38; median=10.35 months) compared to those with intermediate *TRADD* levels (n=142; median 15.8 months; *p*=0.0091). Comparison of survival in glioblastoma patients on the basis of expression of genes involved in intrinsic apoptosis pathway such as **(C)** *BCL2L1* (*p*=0.37) and **(D)** *MCL1* (*p*=0.79) did not demonstrate a correlation with survival.

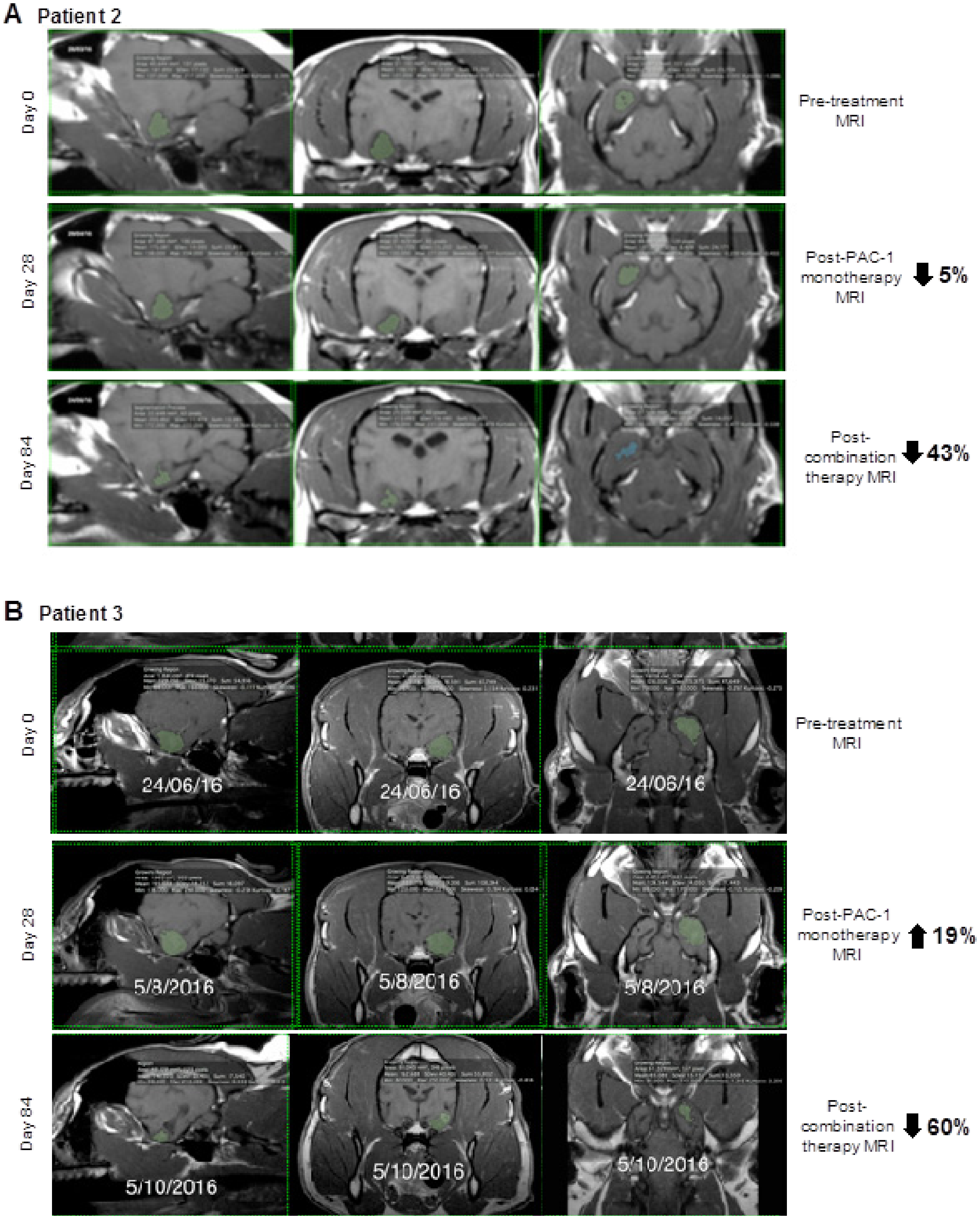

Supplementary Figure 2: Evaluation of combination of oral PAC-1 with TMZ and definitive ionizing radiation therapy in pet dogs with non-resectable gliomas. PAC-1 was administered orally from days 1-84. TMZ was administered orally days 29-33 and 57-61. Shown are MRIs of gliomas of Patient 2 **(A)** and Patient 3 **(B)** on Day 0 (pre-treatment), Day 28 (post PAC-1 monotherapy), and Day 84 (post combination therapy). Tumors have been false-colored green. Change in tumor size from Day 0 is denoted on far right side of images. *Quantification in Table 1.*

Supplementary Table 1: Cytotoxicity of small-molecules against glioma cell lines

Cell line	Cancer type	PAC-1 72 h IC_{50} (μM)	TMZ 72 h IC_{50} (μM)
U87 (H)	Glioblastoma	15.2 ± 2.8	>100
D54 (H)	Glioblastoma	5.5 ± 0.4	65.9 ± 20
U118-MG (H)	Glioblastoma	27.2 ± 2.6	>100
9L (R)	Glioblastoma	5.7 ± 0.5	>100
GL261 (M)	Glioblastoma	1.3 ± 0.2	>100
020913 (H)	Glioblastoma (onco-sphere)	17.7 ± 3.8	>100
JHH-079 (H)	Glioblastoma (onco-sphere)	18.1 ± 3.1	>100

Abbreviations: H, human; R, rat; M, mouse

REFERENCES

1. Putt KS, Chen GW, Pearson JM, Sandhorst JS, Hoagland MS, Kwon JT, Hwang SK, Jin H, Churchwell MI, Cho MH, Doerge DR, Helferich WG, Hergenrother PJ. Small-molecule activation of procaspase-3 to caspase-3 as a personalized anticancer strategy. Nat Chem Biol. 2006; 2(10):543-550.
